# The Co-occurrence of Loneliness and Nicotine Use Among German Adolescents: A Cross-Sectional Analysis

**DOI:** 10.1177/1179173X251372794

**Published:** 2025-09-03

**Authors:** Stephanie Klosterhalfen, Julia Hansen, Reiner Hanewinkel

**Affiliations:** 1 Institute of General Practice (ifam), Centre for Health and Society (chs), Addiction Research, and Clinical Epidemiology Unit, Medical Faculty and University Hospital Düsseldorf, 9170 Heinrich, Heine University Düsseldorf, Düsseldorf, Germany; 2 221536 Institute for Therapy and Health Research, IFT-Nord, Kiel, Germany

**Keywords:** loneliness, youth, smoking, nicotine, Germany

## Abstract

**Introduction:**

Loneliness, a feeling of inadequate social relationships, is associated with behavioural health issues. This study examines the co-occurrence of loneliness and nicotine use (e-cigarettes, cigarettes and waterpipe (WP)) among adolescents aged 9-17 years in Germany.

**Methods:**

Data were derived from the eighth wave of the “Präventionsradar”, an annual school-based survey in Germany. The sample included 23 009 adolescents aged 9-17 years, from 107 schools and 1449 classes, who completed online questionnaires between November 2023 and February 2024. Prevalence rates and multilevel logistic regression models were used to assess associations between loneliness, measured using the three-item UCLA Loneliness Scale, and current nicotine use, adjusting for covariates (age, gender, social status, sensation seeking, school type).

**Results:**

Loneliness was reported by 31.5% of adolescents. It was associated with current cigarette use (OR 1.70, 95% CI 1.43-2.02), e-cigarette use (OR 1.59, 95% CI 1.38-1.83), WP use (OR 1.63, 95% CI 1.25-2.13), and any nicotine product use (OR 1.58, 95% CI 1.39-1.80). Early adolescents (11- to 14-year-olds) experiencing loneliness showed a higher risk of WP use (OR 1.90, 95% CI 1.16-3.13) and nicotine use (OR 1.39, 95% CI 1.07-1.80) compared to middle adolescents (15- to 17-year-olds).

**Conclusions:**

Loneliness is prevalent and significantly linked to nicotine use among German adolescents, highlighting the need to address social factors in nicotine prevention efforts. The cross-sectional design limits causal inference.

## Introduction

Loneliness, *a feeling of perceived isolation,* affects all age groups and spans across the globe, and is defined as a significant public health issue.^1^ Most epidemiological studies of loneliness focus on the older age groups,^2^ but loneliness also occurs during adolescence^3^ and has increased among children and adolescents in recent years.^4^ The prevalence of loneliness varies across studies. In Germany, approximately one-third of the adult population reports feelings of loneliness. Among adolescents in Germany, aged between 11 and 15 years, approximately 13% perceive loneliness.^5,6^ Prevalence of loneliness was found to be significantly higher among girls, older adolescents and children and adolescents from lower socio-economic backgrounds.^7^

The detrimental impact of loneliness on health is becoming increasingly acknowledged.^8^ There is a growing body of evidence indicating that loneliness is associated with an increased risk of several mental health problems, including depression, bad sleep quality, and anxiety. Furthermore, loneliness is linked to social and environmental outcomes, such as bullying victimization and involvement in fighting, as well as health-risk behaviour outcomes, such as smoking.^9,10^

Smoking is a persistent health problem among the population. Studies show that people often start smoking/vaping in adolescence, which increases the risk of long-term nicotine addiction.^11^ In 2021, Germany was ranked 34th out of 36 countries on the Tobacco Control Scale, indicating that measures to reduce tobacco consumption and mitigate the negative impacts of smoking are insufficiently present or inadequately implemented. One consequence of this is the high prevalence of smoking. In 2023, 7.4% of 12-17-year-olds,^12^ and approximately 30% of the entire population (14-99 year-olds)^13^ still smoke cigarettes. In addition to cigarettes, other inhaled nicotine products are available in Germany, including e-cigarettes and WPs (see Supplemental 1).

People who feel lonely are more likely to engage in smoking behaviour.^14^ The findings of a study conducted with an adolescent sample indicated that approximately 45% of respondents who smoked cigarettes reported feelings of loneliness, in contrast to approximately 25% of respondents who had never used a tobacco product.^9^ Although the direction of the association remains controversial — whether loneliness causes or leading to increased smoking^15^ or smoking leads to increased loneliness^15,16^ — has not been conclusively resolved. Over a 12 year follow-up study of older adults in England smoking was associated with increased loneliness over time.^16^ Results of a cross-sectional study showed that people with a high sense of loneliness had a more positive attitude towards cigarette smoking. Students who reported currently smoking cigarettes felt lonelier compared to non-smokers.^17^

Despite the growing body of evidence indicating that loneliness is associated with an increased risk of smoking, there is a paucity of knowledge regarding the association between loneliness and different nicotine product use. A more nuanced comprehension of the association between loneliness and smoking and vaping behaviour may facilitate the development and implementation of intervention strategies.

To the best of our knowledge, this study aims to be the first to examine the associations between loneliness and nicotine use among adolescents aged 11 to 17 years in Germany using a large, representative sample. This age range corresponds to students in lower secondary education and thus provides a relevant population for investigating substance use in a school-based context. Moreover, the data permit a detailed examination of the association between loneliness and smoking/vaping, with particular focus on the use of different nicotine and tobacco inhalation products (cigarettes, e-cigarettes, WPs), while controlling for a range of confounding variables, including age, gender, subjective social status, sensation seeking and school type.

## Methods

### Study Design

The cross-sectional data were obtained from the latest (eighth) waves of the “Präventionsradar”, a school-based observational study conducted on an annual basis in secondary schools in Germany since the 2016/2017 academic year.

All general education schools with a secondary level (grades 5-10) were invited via email to register classes in grades 5-10 for participation via the study website Präventionsradar. The survey was conducted on an annual basis via an online questionnaire during the winter months, from November 2023 to February 2024. The online questionnaires require no longer than 45 minutes to complete. While repeated participation across survey waves is generally permitted, the present analysis is based solely on cross-sectional data from the 8th survey wave: A total of 107 schools consented to participate in the study. Initially, 1696 classes were registered by these schools. Prior to enrolment, 247 classes withdrew, resulting in 1449 classes officially participating. Among these, 23 154 students completed the questionnaire, of whom 23 009 were included in the final analysis. In early summer, at the end of the academic year, schools receive a comprehensive evaluation of the health-related behaviours exhibited by their pupils. The results report provides a foundation for the implementation of needs-based prevention programs and their subsequent evaluation in instances of repeated participation.

Students who belong to a class registered by the school for the study and can provide written parental consent to take part in the survey are eligible to participate. This is issued in advance of the surveys and collected by the teachers.

## Measures

For transparency and reproducibility, the questionnaire is provided in Supplement 2.

## Primary Outcomes

The primary outcomes of the study were current use of (a) cigarettes, (b) e-cigarettes, (c) waterpipes (WP) and (d) nicotine use (defined as use of any of the three nicotine products).

Students were asked how often they currently smoke cigarettes, e-cigarettes, and WPs. The response categories were “not at all,” “less than once a month,” “at least once a month but not every week,” “at least once a week but not every day,” and “every day.” For the purposes of statistical analysis, the responses “not at all” and “less than once a month” were combined and coded as “0”, indicating no use. The other answers (“at least once a month, but not every week”, “at least once a week, but not every day” and “(almost) every day”) were summarised and coded as “1”, indicating use.

### Predictor Variable

Loneliness was assessed using the University of California, Los Angeles (UCLA) Three-Item Loneliness Scale.^18^ The adolescents were asked three questions about loneliness: “How often do you feel left out?”, “How often do you feel that you lack companionship?”, and “How often do you feel isolated from others?” They could choose between the answer options “hardly ever” (1), “sometimes” (2) and “often” (3). The responses were summed to produce a loneliness score ranging from 3 to 9, with a higher score indicating greater loneliness. A dichotomous variable indicating the presence or absence of loneliness was created: those who did not experience loneliness (range 3-5) and those who did experience loneliness (range 6-9).^19^ The dichotomisation of the UCLA Loneliness Scale was based on methodological considerations and informed by empirically supported cut-off values.^20^

### Confounders/Control

Age was assessed as a continuous variable and ranged from 9 to 17 years, including early adolescence from 11 to 14 years and middle adolescence from 15 to 17 years.^21^ The variable of gender was assessed as male, female, or diverse and subsequently categorized as binary (0 = female, 1 = male) in the regression models. This was done due to the relatively small size of the diverse group, which constituted only 1.8% of the total sample. School type was categorized as 0 = other and 1 = Gymnasium. The Gymnasium is the most advanced type of secondary school in Germany, with a strong emphasis on academic learning. Perceived social status was measured using the MacArthur Scale,^22^ which has been demonstrated to have medium correlations with single objective SES indicators.^23^ Scale ranges from 1 to 10 (high). Sensation seeking, which is central to the prevention of risky health behaviours, was measured with a brief two-item scale.^24^ Scale ranges from 1 to 5 (high).

### Statistical Analyses

Data analysis was performed using STATA, Version 17.0 (Stata Corp, College Station, Texas, USA).

To examine the associations between loneliness and nicotine use, adjusted multilevel logistic regression models were constructed. Given the nested nature of the data, we included students’ school class as a level 2 variable within the models. The adjusted models controlled for the effect of age (continuous variable), gender (dichotomous variable: male (1)/female (0)), school type (dichotomous variable: gymnasium (higher academic level; 1) and other schools (0)), sensation seeking (dichotomous variable: low (0)/high (0)) and perceived social status (dichotomous variable: low (0)/high (1)) on the dependent variables. For testing interactions, we defined two age categories (early adolescence (1)/middle adolescence (0)). Children (age 9 and 10) were excluded from the analyses because current nicotine use was not present in the age group.

Adjusted Odds Ratio (OR), 95% Confidence intervals (CI), and *P*-values were reported. Statistical significance set at *P* < .05. Missing data were handled by listwise deletion. To adjust for biases in representativeness, the data were weighted to census data. For more information, see Präventionsradar-weighting.

## Results

The present study included 23 009 adolescent participants in the total sample, comprising males, females and diverse gender identities, aged between 9 and 17 years old, who provided responses on their experience of loneliness. The mean age was 13.2 years (SD = 1.74) and 47.8% were male gender. The respondents came from 107 different schools and 1449 classes in grades 5 to 10. All participants filled out the questionnaire between November 2023 and February 2024 in their schools.

The prevalence of loneliness was 31.5% (95% CI 30.8-32.1) among German adolescents aged 9 to 17 years. Gender-specific differences were observed, with females reporting a higher prevalence of loneliness (39.0% (95% CI 38.1-40.0)) compared to males (23.7% (95% CI 22.8-24.6)). Table 1 shows covariates and their relationship to loneliness. Experiencing loneliness was higher in lower subjective social status, having higher sensation seeking/rebelliousness, and with school type other than gymnasium. Loneliness was observed to be consistent across all age groups.

**Table 1.** Covariates and Their Relationship to Loneliness

Parameter	Loneliness	
	Yes	No
Categorical variables, n (row %)		
No. of adolescents	7232 (31.5)	15 772 (68.5)
Age, y		
9-10	407 (30.8)	990 (69.2)
11	847 (29.7)	2074 (70.3)
12	1206 (31.8)	2641 (68.2)
13	1355 (31.7)	2942 (68.3)
14	1299 (31.2)	2798 (68.8)
15	1307 (32.4)	2652 (67.6)
16-17	726 (31.4)	1537 (68.6)
Gender		
female	4475 (39.0)	7098 (61.0)
male	2411 (23.7)	8167 (76.3)
School type		
Gymnasium	4303 (29.5)	9916 (70.5)
other	2934 (33.0)	5856 (67.0)
Subjective social status		
high	14 718 (30.3)	6440 (69.6)
low	547 (48.0)	516 (52.0)
Sensation seeking		
high	1000 (36.5)	1745 (63.5)
low	6210 (30.7)	13 983 (69.3)

Loneliness is defined as a UCLA scale range of 6-9, whereas no loneliness is defined as a scale range of 3-5. Low subjective social status is defined as a MacArthur scale range from 1-4, else is defined as high. High sensation seeking is defined as scale range of 4-5, else is defined as low.

### Associations of loneliness with current smoking of e-cigarettes, cigarettes, WPs and nicotine use

Associations between loneliness and current use of nicotine products were observed after adjustment for covariates. Adolescents experiencing loneliness were more likely to smoke e-cigarettes (OR 1.59, 95% CI 1.38-1.83, *P* < .001), cigarettes (OR 1.70, 95% CI 1.43-2.02, *P* < .001), WP (OR 1.63, 95% CI 1.25-2.13, *P* < .001) or use any nicotine product (OR 1.58, 95% CI 1.39-1.80, *P* < .001) than those without perceived loneliness.

**Figure 1.**
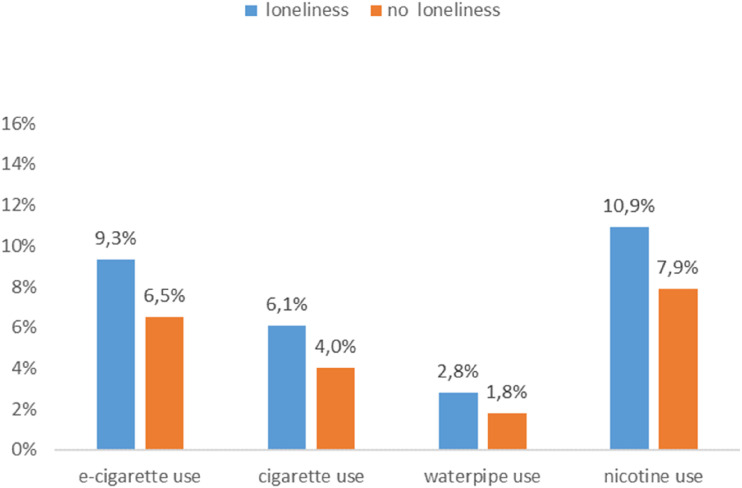
Loneliness and Current (Monthly) Use of e-Cigarettes, Cigarettes, WP Use, and Nicotine (Defined as Use of Any of the Three Nicotine Products); Adjusted Prevalence for Current Use after Controlling for Potential Confounding Factors including as Age, Gender, School Type, Sensation Seeking, Subjective Social Status

The estimated prevalence of nicotine uses in the presence of loneliness, after controlling for confounding factors (age, gender, school type, sensation seeking, and subjective social status) is 9.3% (95% CI 8.5-10.0) for e-cigarette use and 6.1% (95% CI 5.4-6.7) for cigarette use. The prevalence of WP use was 2.8% (95% CI 2.3-3.3), while the prevalence of nicotine use (defined as smoking at least one out of three products) was 10.9% (95% CI 10.1-11.8), see Figure 1.

### Loneliness and Nicotine Use in Early and Middle Adolescence

**Figure 2.**
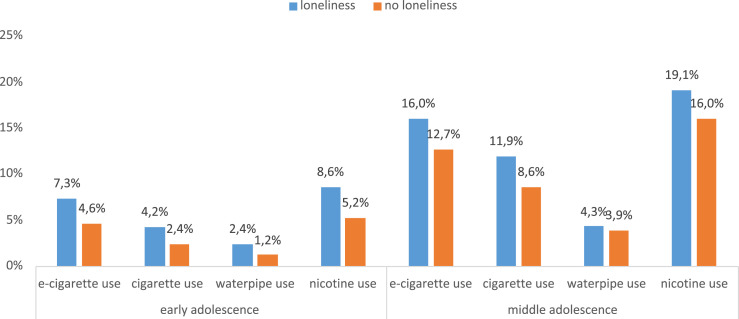
Loneliness x Stage of Adolescence and Current (Monthly) Use of e-Cigarettes, Cigarettes, WP Use, and Nicotine. Nicotine Use Defined as Use of Any of the Three Nicotine Products; Adjusted Prevalence for Current Use after Controlling for Potential Confounding Factors including as Age, Gender, School Type, Sensation Seeking, Subjective Social Status

Lonely adolescents, both in early and middle adolescence, are more likely to use nicotine and tobacco products than their non-lonely peers. The estimated prevalence of nicotine use in the presence of loneliness, after controlling for confounding factors (age, gender, school type, sensation-seeking and subjective social status), is 8.6% (95% CI 7.5-9.6) in early adolescence and 19.1% (95% CI 17.2-21.1) in middle adolescence. In both cases, the prevalence is higher than in the non-lonely comparison groups. This applies to all nicotine products analysed (see Figure 2).

Testing for interaction effects found significant effect modifications related to loneliness x stage of adolescence and current WP (OR 1.90, 95% CI 1.16-3.13, *P* = .011) and nicotine use (OR 1.39, 95% CI 1.07-1.80, *P* = .012). Early adolescence is associated with greater risk of WP use and nicotine use in the presence of loneliness than middle adolescence. Results for cigarette and e-cigarette use are in the same direction but statistically not significant.

## Discussion

The data from this observational study with a large sample of more than 23 000 German adolescents indicate that about one third of adolescents reported an increased burden of loneliness and that there is an association between loneliness and current use of nicotine products in Germany. Loneliness is associated with a greater likelihood of using nicotine products, irrespective of the specific product in which the nicotine is delivered. Loneliness in early adolescence is a risk factor for current use of WPs or any nicotine product.

Notably, there are pronounced gender differences, with females experiencing a higher prevalence of loneliness (39.0%) compared to males (23.7%). These findings align with previous research suggesting that adolescent females may be more vulnerable to feelings of loneliness due to social and emotional factors.^7,25^ Align with further international studies,^26^ our data also indicate a concerning co-occurrence of loneliness and nicotine use among adolescents, even after adjusting for potential confounders. Adolescents experiencing loneliness were significantly more likely to engage in the use of e-cigarettes, cigarettes, WPs, or any nicotine product compared to their non-lonely peers. The odds ratios for these behaviors indicate a robust association, with lonely adolescents showing a heightened propensity for nicotine use, particularly for smoking WPs (OR 1.63) and cigarettes (OR 1.70). These findings are concerning as they suggest that loneliness may serve as a catalyst for nicotine use among adolescents, potentially as a coping mechanism for their social and emotional distress. Several theoretical frameworks may help explain the association between loneliness and nicotine use observed in this study. One explanation is the self-medication hypothesis, which posits that adolescents experiencing emotional distress such as loneliness may turn to nicotine to alleviate negative affect or improve mood.^27^ Similarly, nicotine use may act as a coping mechanism, providing temporary relief from feelings of social disconnection or psychological strain.^28^ From a social facilitation perspective, adolescents might use nicotine to gain acceptance or belonging within peer groups, especially when facing loneliness or social exclusion.^29^ These mechanisms may operate simultaneously and interact in complex ways. Future longitudinal and qualitative studies should investigate these pathways and clarify the directionality of associations. The study also identified other important factors associated with loneliness, including higher sensation-seeking behaviour.^30^ These factors, combined with nicotine use behaviour, create a nuanced picture of the adolescent who perceived feeling lonely. Studies have shown that people with low socioeconomic status are particularly affected by loneliness,^5,7,25^ which is also the population group with the highest smoking prevalence in Germany. Interestingly, the consistency of loneliness across different age groups implies that this issue is pervasive throughout adolescence, not confined to a specific developmental stage.

Importantly, the study finds that the relationship between loneliness and nicotine use is moderated by the stage of adolescence. Early adolescents who experience loneliness are at a greater risk of WP and nicotine use than their middle-adolescent counterparts.^31^ The significant interaction effects between loneliness and stage of adolescence (OR=1.90 for WP use and OR=1.39 for overall nicotine use) suggest that early adolescence is a particularly critical period where interventions to prevent nicotine use may be most effective.^32^ While the trends for cigarette and e-cigarette use follow a similar pattern, they do not reach statistical significance, indicating that the influence of loneliness may be more pronounced for certain types of nicotine products, such as WP, during early adolescence. WP smoking, often perceived as a social activity, may attract both lonely individuals seeking social interaction and those who are socially well-connected. A study from Iran in 2019 suggests a potentially strong connection between WP consumption and loneliness^33^: In this cross-sectional, questionnaire-based study conducted with 900 participants who currently smoke WPs (aged 18 and older), ‘Escape from loneliness’ was among the top three reasons cited for initiating WP smoking. Another hypothesis of the study team is, that people who smoke WPs alone, rather than in a group setting, are more likely to experience loneliness. This hypothesis could be further investigated if data on whether smoking occurs alone or in a group were available.

The strong relationship between loneliness and nicotine use smoking highlights that as loneliness intensifies, the likelihood of nicotine use increases, making loneliness a key target for preventive interventions. This is further supported by a recent Cochrane systematic review, which suggests that smoking cessation is associated with improvements in anxiety, depression, social well-being, and social isolation. Together, these findings indicate that addressing loneliness can not only reduce the risk of nicotine use but also contribute to improvements in mental and social health.^34^

Furthermore, loneliness is a growing societal problem that negatively impacts both the mental and physical health of those affected. In response, the German government has developed a strategy against loneliness in 2021, which includes measures to promote social networks, improve access to social services, raise public awareness, support research, and collaborate with various stakeholders.

## Strengths and Limitations

The study exhibits several strengths, including a large sample size and a focus on a young target population, which enhances the generalizability of the findings. Additionally, the inclusion of diverse school types provides a comprehensive overview of different educational environments. Systematic selection bias may have occurred due to the voluntary nature of participation in the study, which was addressed by calculating a weighting factor based on the German Federal Statistical Office. A key strength is the ability to differentiate between loneliness and various forms of smoking behaviour, such as cigarette, e-cigarette, and WP use, allowing for a nuanced analysis among adolescents.

However, the study also has certain limitations. It lacks detailed information about WP consumption, specifically whether it occurs alone or in groups, which could influence the social dynamics of smoking behaviour. Furthermore, the study design does not permit causal inferences, limiting the ability to establish cause-and-effect relationships. While it is plausible that loneliness increases the likelihood of nicotine use, reverse causality cannot be ruled out — for instance, nicotine use may lead to increased social isolation or feelings of loneliness. Moreover, recent evidence suggests that the relationship between loneliness and substance use may be bidirectional, with each reinforcing the other over time. This dynamic interplay underscores the importance of longitudinal research to disentangle causal pathways and to identify potential windows for intervention. Nevertheless, one argument supporting the hypothesised direction is that substance use in adolescence often occurs in social contexts and within peer groups, suggesting that a lack of social integration (ie, loneliness) may precede and contribute to substance use initiation. In addition, the voluntary participation of schools may affect the generalisability of the findings. Schools that chose to take part may differ systematically from those that did not — for example, in terms of socio-demographic characteristics, school environment, or openness to health-related topics. As a result, the sample may not fully represent the broader adolescent population. The influence of migration on feelings of loneliness and smoking behaviour, is another area where more data is needed. Moreover, data collection was conducted only during the winter months, which might have resulted in higher reported levels of loneliness compared to other seasons, such as summer. Additionally, as the data are entirely self-reported through questionnaires, there is a risk of social desirability bias and other external influences that may affect the accuracy of the responses. While most of the scales used in this study were based on previously validated instruments, not all questionnaire items were formally validated in this specific age group. This represents a limitation and should be considered when interpreting the results. However, the use of widely established measures, together with the large and diverse sample, strengthens the overall reliability and generalisability of the findings. One further limitation of this study is the absence of an a priori power analysis to justify the sample size. As the study examining the association between loneliness and smoking was based on a large convenience sample drawn from a pre-existing dataset, no formal calculation was performed prior to analysis. However, the dataset was weighted and adjusted to reflect the underlying population structure, which enhances the representativeness of the findings. It should also be noted that in large-scale observational studies such as this one, the need for an a priori power calculation is generally less critical, as the extensive sample size typically provides sufficient statistical power to detect meaningful effects. Nonetheless, the absence of a formal power analysis should be considered when interpreting the statistical precision of the results.

## Conclusion

In summary, loneliness is a critical factor in adolescent nicotine use, particularly for traditional tobacco products. Addressing loneliness could be a key strategy in reducing smoking rates among this population. Future research should explore targeted interventions that address both loneliness and nicotine use in adolescents, particularly those in early adolescence, to mitigate the potential for nicotine dependence and its associated health risks. Comprehensive prevention programs are needed that not only provide information about the health risks of smoking/vaping, but also show alternative ways of social integration.

## Supplemental Material

Supplemental Material - The Co-occurrence of Loneliness and Nicotine Use Among German Adolescents: A Cross-Sectional Analysis Supplemental Material for The Co-occurrence of Loneliness and Nicotine Use Among German Adolescents: A Cross-Sectional Analysis by Stephanie Klosterhalfen, Julia Hansen, Reiner Hanewinkel in Tobacco Use Insights.

Supplemental Material - The Co-occurrence of Loneliness and Nicotine Use Among German Adolescents: A Cross-Sectional Analysis Supplemental Material for The Co-occurrence of Loneliness and Nicotine Use Among German Adolescents: A Cross-Sectional Analysis by Stephanie Klosterhalfen, Julia Hansen, Reiner Hanewinkel in Tobacco Use Insights.

## Data Availability

The data that support the findings of this study are available from the corresponding author upon reasonable request.*
