# Supplementary material for: The Co-occurrence of Loneliness and Nicotine Use Among German Adolescents: A Cross-Sectional Analysis
Source: Tob Use Insights. 2025 Sep 3;18:1179173X251372794. doi: 10.1177/1179173X251372794 (PMC12409023; doi:10.1177/1179173X251372794)
Supplement: Supplemental Material - The Co-occurrence of Loneliness and Nicotine Use Among German Adolescents: A Cross-Sectional Analysis [file sj-pdf-1-tui-10.1177_1179173X251372794.pdf]

Supplementary 1 Frequently used nicotine products in Germany – an overview

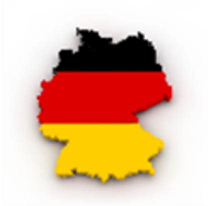

	Cigarette	E-cigarette	Waterpipe
	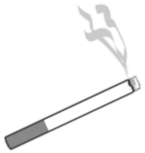	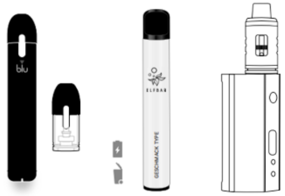	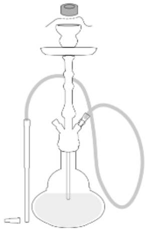
Permission age [1]	18 +	18 +	18 +
Prevalence (12-17-year olds)<br>Trend (reference 2021) [2]*	7.4% ↗	3.9% ↗	3.9% ↘
Prevalence (18-25-year olds)<br>Trend (reference 2021) [2]*	25.4% ↘	7.8% ↗	10.2% ↘
Flavours, like fruity permitted [3]	no	yes	yes
Function/ Specialities	• During smoking tobacco burns at about 900°Celsius<br>• Most widespread<br>• Relatively cheap	• Electronic vaporising of e-liquid<br>• Different types: disposables, pods, tanks<br>• Disposables: about 600 puffs<br>• Later market entry in 2007	• Inhalation of water-cooled smoke<br>• Multiple hoses connectable<br>• Special locations (shisha bar)<br>• Duration of a session up to 90 minutes

References:

[1] Jugendschutzgesetz (JuSchG). Jugendschutzgesetz vom 23. Juli 2002 (BGBl. I S. 2730), das zuletzt durch Artikel 2 des Gesetzes vom 22.Oktober 2020 (BGBl. I S. 2229) geändert worden ist. 28.12.2020. Retrieved 22.04.2024 from: https://www.gesetze-im-internet.de/juschg/__10.html

[2] *current use; Bundeszentrale für gesundheitliche Aufklärung. Die Drogenaffinität Jugendlicher in der Bundesrepublik Deutschland 2023 [Internet]. 2024. Retrieved 04.07.2024 from: https://www.bzga.de/presse/daten-und-fakten/suchtpraevention

[3] Gesetz über Tabakerzeugnisse und verwandte Erzeugnisse (Tabakerzeugnisgesetz - TabakerzG) [Internet]. 2016. Retrieved 20.06.2024 from: https://www.gesetze-im-internet.de/tabakerzg/TabakerzG.pdf
