# Supplementary material for: The Co-occurrence of Loneliness and Nicotine Use Among German Adolescents: A Cross-Sectional Analysis
Source: Tob Use Insights. 2025 Sep 3;18:1179173X251372794. doi: 10.1177/1179173X251372794 (PMC12409023; doi:10.1177/1179173X251372794)
Supplement: Supplemental Material - The Co-occurrence of Loneliness and Nicotine Use Among German Adolescents: A Cross-Sectional Analysis [file sj-pdf-2-tui-10.1177_1179173X251372794.pdf]

Questionnaire

- I am… choices: {1: 'male', 2: female', 3: 'diverse'}
- Please enter your age here.
- Imagine a ladder with ten rungs that represents where people in Germany stand. Where would you place your family on this ladder; choices: {10: ' Most money, best jobs, highest level of education, 9: '9', 8: '8', 7: '7', 6: '6', 5: '5', 4: '4', 3: '3', 2: '2', 1: Least money, worst jobs, lowest level of education'} [1-3]
- How often do you do dangerous things just for fun; How often do you do exciting things, even if they are dangerous? choices: {1: 'Not at all', 2: 'Occasionally', 3: 'Sometimes', 4: 'Often', 5: 'Very often'} [4]
- 'How often do you feel alone? '; 'How often do you feel left out? '; 'How often do you feel like you are missing friends? '; choices: {1: Hardly ever, 2: Sometimes, 3: Often} [5-6]
- 'How often do you currently smoke cigarettes?'; 'How often do you currently smoke e-cigarettes?'; 'How often do you currently smoke shishas (waterpipe)?'; choices: {1: 'Not at all', 2: 'Less than once a month', 3: At least once a month, but not every week', 4: 'At least once a week, but not every day', 5: '(Almost) every day'}

  Monthly use is the standard indicator applied in relevant international studies, such as the Global Youth Tobacco Survey (GYTS), and is also used by the CDC. [7-9]

1. Goodman E, Adler NE, Kawachi I, Frazier AL, Huang B, Colditz GA. Adolescents' perceptions of social status: development and evaluation of a new indicator. *Pediatrics*. 2001;108(2):E31.

2. Hoebel J, Müters S, Kuntz B, Lange C, Lampert T. Messung des subjektiven sozialen Status in der Gesundheitsforschung mit einer deutschen Version der MacArthur Scale. *Bundesgesundheitsblatt Gesundheitsforschung Gesundheitsschutz*. 2015;58(7):749-757.

3. Lampert T, Hoebel J, Kuntz B, Müters S, Kroll LE. Messung des sozioökonomischen Status und des subjektiven sozialen Status in KiGGS Welle 2. In: *Beiträge zur Gesundheitsberichterstattung des Bundes*. Vol 3. Berlin: Robert Koch-Institut, Epidemiologie und Gesundheitsberichterstattung; 2018.

4. Stephenson MT, Hoyle RH, Palmgreen P, Slater MD. Brief measures of sensation seeking for screening and large-scale surveys. *Drug Alcohol Depend*. 2003;72(3):279-286.

5. Gosling CJ, Colle R, Cartigny A, Jollant F, Corruble E, Frajerman A. Measuring loneliness: a head-to-head psychometric comparison of the 3- and 20-item UCLA Loneliness Scales. *Psychol Med*. 2024;54(14):1-7.

6. Trucharte A, Calderón L, Cerezo E, Contreras A, Peinado V, Valiente C. Three-item loneliness scale: psychometric properties and normative data of the Spanish version. *Curr Psychol*. 2023;42(9):7466-7474.

7. Sreeramareddy CT, Acharya K, Manoharan A, Oo PS. Changes in E-cigarette Use, Cigarette Smoking, and Dual-Use Among the Youth (13–15 Years) in 10 Countries (2013–2019): Analyses of Global Youth Tobacco Surveys. *Nicotine Tob Res*. 2024;26(2):142-150.

8. Sreeramareddy CT, Acharya K, Manoharan A. Electronic cigarettes use and 'dual use' among the youth in 75 countries: estimates from Global Youth Tobacco Surveys (2014–2019). *Sci Rep*. 2022;12(1):20967.

9.Centers for Disease Control and Prevention (CDC). Tobacco Product Use Among Youths — United States, 2023. MMWR Morb Mortal Wkly Rep. 2023;72(44):1177-1183. Accessed August 7, 2025. https://www.cdc.gov/mmwr/volumes/72/wr/mm7244a1.htm
